# A Numerical Study on Optimization of Shape and Dimensions for Cold-Extruded Blank of Copper Pin-Type Heat Dissipation Substrates

**DOI:** 10.3390/ma19050962

**Published:** 2026-03-02

**Authors:** Wei Wei, Fakai Chen, Jingbo Gao, Yong Xu, Tengfei Zhang, Wenlong Xie

**Affiliations:** 1 Zhejiang Hailiang Co., Ltd., Zhuji 311835, China; ww857821205@126.com (W.W.); gaojingbo@hailiang.com (J.G.); soar95@163.com (T.Z.); 2 College of Metallurgy and Energy, North China University of Science and Technology, Tangshan 063210, China; fkchen0301@163.com; 3 Institute of Metal Research, Chinese Academy of Sciences, Shenyang 110016, China; 4 School of Materials Science and Engineering, Harbin Institute of Technology at Weihai, Weihai 264209, China

**Keywords:** heat sink substrates, cold extrusion, blank shape optimization

## Abstract

The thermal dissipation performance of the radiator is crucial for the stable operation of power electronic devices. Due to excellent thermal performance, copper pin-type heat sink substrates are widely adopted. However, the cold extrusion process for heat sink substrates suffers from low material utilization and high forming loads. To improve material utilization and reduce cold extrusion forming load, four blank shapes (rectangular, trapezoidal, trapezoidal cap, and stepped) were designed using finite-element simulation to investigate the effects of blank shape and placement method with orientation relative to the die cavity on forming quality. Further dimensional optimization was conducted to determine the optimal configuration. The results show that the stepped blank with front orientation exhibits the optimal forming performance, featuring the lowest forming load and the most sufficient pin-fin filling. Compared with back orientation, front orientation achieves higher and more uniform material flow velocity, and significantly reduces forming load. Through dimension optimization, the 7 mm-thick stepped blank is determined as the optimal solution, with the forming load reduced to 15,000 kN (a 35.3% decrease compared to the initial 7.5 mm stepped blank), and both the substrate thickness and pin-fin height meet the design requirements (4.5 mm and 6.5 mm). Experiments verify the feasibility of the optimized scheme, providing technical support for the low-cost, high-quality mass production of copper pin-type heat sink substrates.

## 1. Introduction

As electronic devices become increasingly high-performance, compact, and lightweight, thermal management has emerged as a critical challenge limiting their stable operation. For instance, if internal chips cannot dissipate heat promptly, their surface temperatures will rise rapidly, and elevated temperatures significantly accelerate degradation mechanisms [1,2].

Heat sink design and optimization are crucial for enhancing equipment reliability and performance. Pin-type heat sink substrates, featuring pin-type structures that significantly increase heat dissipation surface area, effectively enhance module thermal performance, and are widely used in various electronic devices [3,4,5]. Zhou et al. [6] employed a multi-objective genetic algorithm to optimize the geometric parameters of the columns, achieving a 14.2% reduction in thermal resistance under forced convection conditions. However, their study was based on ideal geometric assumptions and did not consider forming-induced geometric deviations, resulting in partial deviations in the local thermal conductivity. The conical column structure proposed by Ahmadian-Elmi et al. [7] demonstrated a 37% pressure drop advantage in simulations. However, the designed structure is difficult to fabricate using conventional processes, and laser additive manufacturing prototypes exhibit high porosity, leading to reduced heat transfer efficiency. Kuppusamy et al. [8] introduced slanted flow passages within the channels to generate secondary flow. The results indicated that the optimized model exhibited a 146% improvement in overall performance and a 76.8% reduction in thermal resistance compared to the original model. Lin et al. [9] proposed a wavy microchannel heat sink optimized by varying the wavelength and amplitude along the flow direction. Compared with the original design, the optimized heat sink demonstrated a smaller temperature difference on the bottom wall and a lower thermal resistance. Chai et al. [10] designed microchannel heat sinks with five types of ribs arranged along the flow direction and investigated their thermal-hydraulic performance in an interrupted chamber.

Regarding substrate material selection, options typically include metal substrates, ceramic substrates, and composite substrates. Ceramic materials offer excellent insulation properties and high-temperature stability, maintaining consistent performance in high-temperature environments. They are suitable as substrate materials for high-voltage, high-frequency, and high-power devices. Zou et al. [11] investigated the thermal performance of AlN ceramic substrates in high-power light-emitting diodes, finding superior heat dissipation compared to Al_2_O_3_ substrates. Tsao et al. [12] packaged GaN chips on both traditional RO3010 laminate and AlN substrates. They observed that chips on RO3010 substrates exhibited a linear increase in operating temperature with power, whereas chips on AlN substrates showed a smaller temperature variation. Despite the numerous advantages of ceramic substrates, their high brittleness makes them prone to fracture under impact, limiting their application. Composite substrate heat dissipation technology involves combining high thermal conductivity materials with other materials through specialized processes to form packaging substrates with excellent thermal performance. For instance, graphene can be incorporated into ceramic, metallic, and polymeric materials to create graphene-reinforced composite substrates [13]. Rho et al. [14] proposed a synthetic porous copper-graphene heterostructure that significantly enhances thermal conductivity. Chen et al. [15] synthesized a reduced GO (rGO) sol coating and regarded it as TIM for WLEDs. Liang et al. [16] proposed a novel packaging strategy to reduce the interface thermal resistance of surface-mounted ultraviolet LED chips based on a silicone with rGO as a solder filler. However, composite substrate processing presents challenges, including high difficulty, elevated costs, and interface strength issues.

Metal materials were the earliest materials applied for substrate heat dissipation. In particular, commonly used metals such as silver, copper, iron, and aluminum are widely selected as packaging substrates for electronic devices, owing to the mechanical strength required for heat sink applications and favorable machinability. Although the thermal conductivity of silver (~429 W/(m·K)) is slightly higher than that of copper (~401 W/(m·K)), the high price of silver severely limits its practical application. Iron and aluminum are inexpensive but exhibit significantly lower thermal conductivity than copper. Copper, with its excellent thermal properties, remains the most prevalent material for fin-type heat sink substrates. However, its cost remains relatively high, necessitating maximized material utilization. Methods for forming metal heat sink substrates include machining, die casting, metal additive manufacturing, metal injection molding, and extrusion. Xiang et al. [17] designed and fabricated a novel phase-change heat sink for high-power LEDs. The manufacturing method involves the following process: with a predetermined stamping depth, the cutting tool moves downward to stamp the workpiece, thereby forming a radial groove. Machining causes high material removal and low utilization for copper pin-fin heat sinks, with thin pin fins prone to deformation and low efficiency, unfit for mass production. Die casting is limited by copper’s high melting point, easily inducing internal defects like porosity and shrinkage that reduce thermal conductivity and compromise pin-fin forming precision. Haga and Fuse [18,19] overcame the limitations of conventional die casting in producing high aspect ratio thin-walled fins (0.5 mm thick) by optimizing the die casting process and employing a highly fluid Al-25%Si alloy. Careri et al. [20] highlighted that metal additive manufacturing technology offers significant advantages in manufacturing compact heat exchangers and complex thin-walled flow channels for aerospace applications. Kaur and Singh [21] conducted an in-depth study on the influence of additive manufacturing surface roughness on the flow and heat transfer characteristics of heat sinks. However, metal additive manufacturing of pure copper heat sinks suffers from high laser reflectivity and unstable molten pools, leading to high part porosity that impairs thermal performance, along with high cost and low efficiency for mass production. Metal injection molding has large sintering shrinkage and poor pin-fin dimensional control, coupled with high requirements for raw powder, cumbersome processes, and insufficient sintered density, which weakens heat dissipation performance. Timelli et al. investigated the fatigue reliability of die-cast aluminum heat sinks under thermal cycling [22], while Singh et al. applied Buckingham’s π theorem to control defects in cold-chamber die casting [23]. Hot extrusion is suitable for constant-cross-section products such as CPU heat sinks [24,25]. Cold extrusion, which forms solid aluminum blocks under high pressure at room temperature, produces heat sinks with lower contact thermal resistance and a thermal conductivity of 226 W/(m·K), outperforming die-cast counterparts [26]. Therefore, extrusion not only achieves high production efficiency but also ensures dimensional accuracy and surface quality of the heat sink substrate. It offers higher material utilization and significantly reduces production costs compared to other forming methods, making it the optimal choice for heat sink substrate fabrication.

This study investigates a copper pin-type heat sink substrate. Aiming to enhance material utilization and reduce cold extrusion tonnage, four distinct blank shapes—rectangular, trapezoidal, trapezoidal cap, and stepped—were proposed. The effects of different blank shapes, dimensions, and placement orientations on cold extrusion outcomes were examined using finite-element simulation. Based on the results, the most suitable shape was determined for testing, providing support for subsequent low-cost, high-quality mass forming of the product.

## 2. Target Parts and Research Proposal

### 2.1. Target Part Dimensions

**Figure 1:**
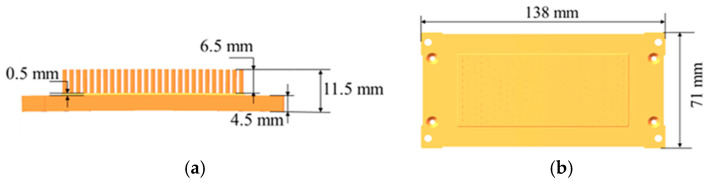
Dimensions of copper pin-type heat spreader substrate: (**a**) side view; (**b**) top view.

Figure 1 shows the dimensions of the copper pin-type heat sink substrate. Rational substrate thickness and pin-fin height not only meet the structural design requirements of the heat sink but also directly determine its thermal dissipation performance and cold extrusion forming feasibility. This component primarily consists of a base plate and pin fins. The total height of the copper substrate is 11.5 mm, with the pin fins measuring 6.5 mm in height. The substrate thickness is 4.5 mm. A small 0.5 mm high boss is located at the base of the pin fins. The overall dimensions are 138 mm × 71 mm. Features such as assembly holes around the perimeter are to be achieved through subsequent machining.

### 2.2. Forming Methods and Equipment

Cold extrusion forming is employed, wherein a metal blank is placed within the cavity of a cold extrusion die. Pressure is applied via extrusion equipment, causing the metal material to undergo plastic flow within the die. This process fills the fin cavities of the fin die, ultimately forming a fin structure with defined shape and dimensions.

**Figure 2:**
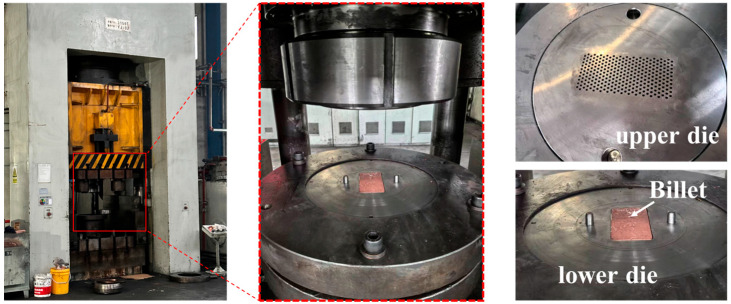
Cold extrusion forming equipment and dies.

The extrusion test was conducted using a 3150 T press from a company in Yancheng, as shown in Figure 2. The die consists of an upper die and a lower die. The cavity shape of the upper die matches the geometric configuration of the needle fins, incorporating detailed features such as needle arrangement, spacing, length, and diameter. The forming process was achieved by limiting the displacement of the upper die, with the tonnage exerted during extrusion recorded by a pressure monitor. A bottom ejection system was designed to push the finished part out of the cavity using a pusher block after extrusion completion. The extrusion process was carried out at room temperature with an extrusion speed of 3 mm/s. The lubricant used was lubricating oil—Fuchs 1905 L.

### 2.3. Finite-Element Model

**Figure 3:**
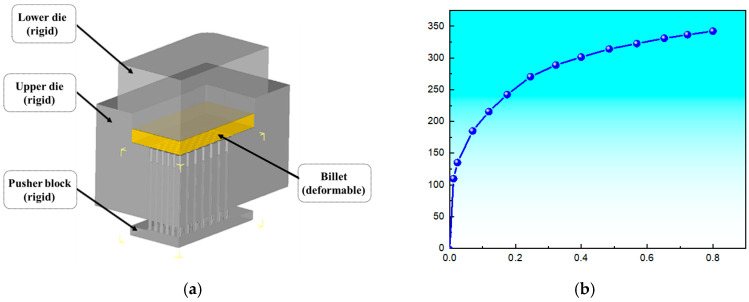
(**a**) 3D finite-element model; (**b**) True stress-strain curve of T2 copper.

The copper pin-type heat sink substrate is symmetrical. To reduce simulation time, a quarter-model was employed for finite-element modeling, as shown in Figure 3a, and imported into the 2023 version of Deform-3Dvia STL format. Two symmetry planes were defined for symmetry constraints. The plate was modeled as a deformable body and refined with a tetrahedral mesh of 2.8 × 10^5^ elements. Based on the maximum and minimum mesh sizes, the solution step size was set to 0.1 mm/step. The die displacement option was selected, with the sparse matrix method and Newton–Rasen iteration chosen for the solver. The simulation temperature was set to 30 °C, and the global friction coefficient was set to 0.12. T2 copper from the Deform material library was selected. Figure 3b shows the true stress-strain curve for T2 copper.

### 2.4. Research Proposal

**Figure 4:**
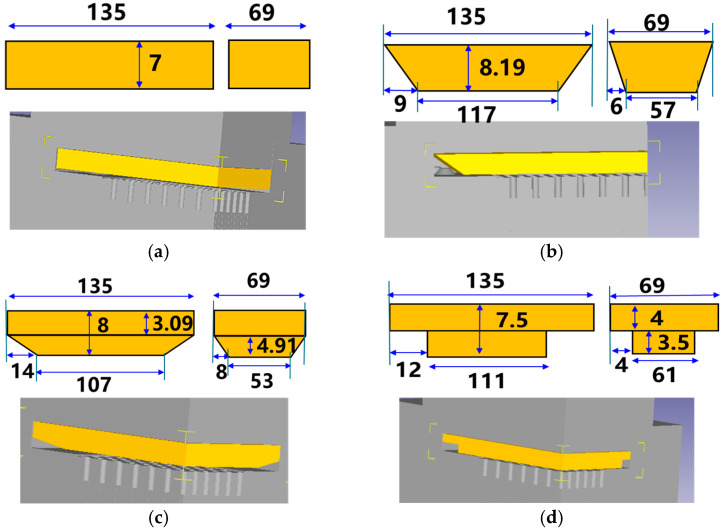
Blank cross-sectional shapes and dimensions: (**a**) rectangular; (**b**) trapezoidal; (**c**) trapezoidal cap; (**d**) stepped.

Based on the overall contour characteristics of the formed part, the initial blank shape was readily considered to be rectangular. After reasonably adding allowances according to the formed part’s volume, a rectangular prism blank measuring 135 × 69 × 7 mm was designed. To investigate the effects of different blank shapes, three additional initial blank shapes were designed while maintaining the same volume as the rectangular blank, as shown in Figure 4. The cross-sectional shapes of the blanks are: (a) rectangular, (b) trapezoidal, (c) trapezoidal cap, and (d) stepped. The specific dimensions of these cross-sectional blanks are also illustrated in Figure 4.

**Figure 5:**
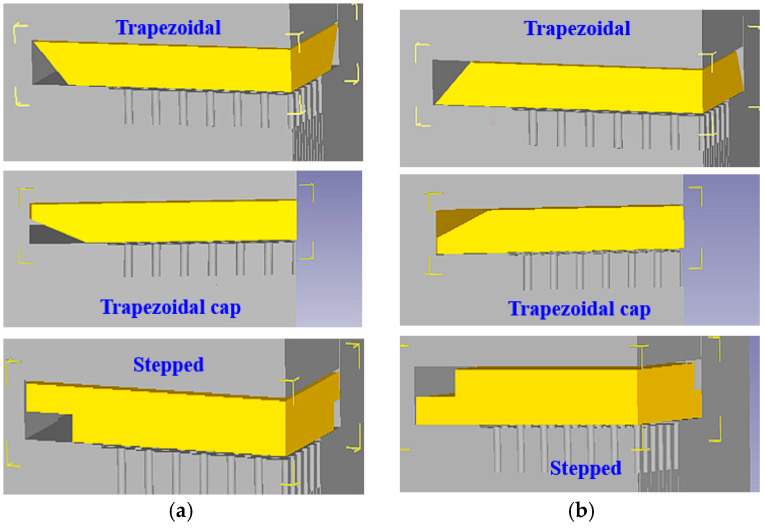
Front and back placement methods for blanks: (**a**) front orientation; (**b**) back orientation.

In addition to blanks with rectangular cross-sections, the other three cross-sectional shapes feature a larger and a smaller area in the thickness direction. When the smaller area faces the needle-fin die, it is considered the front orientation. Figure 5 illustrates the front and back orientations for trapezoidal, trapezoidal cap, and stepped shapes.

## 3. Results

### 3.1. Influence of Initial Shape of the Blank on Forming Quality

**Figure 6:**
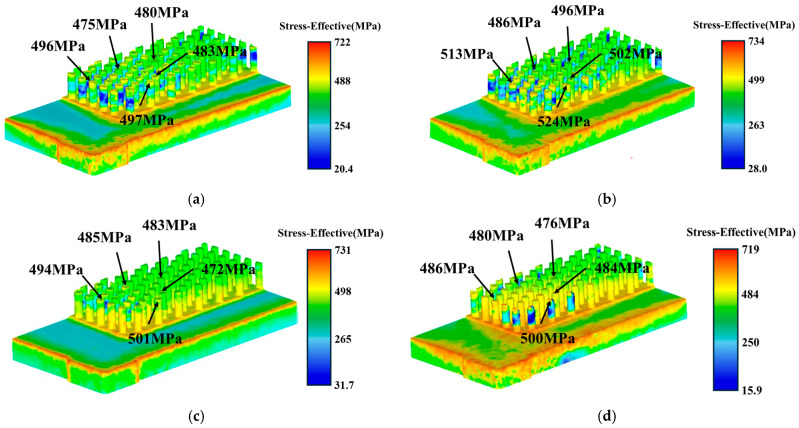
Equivalent stress after forming with different initial shapes: (**a**) rectangular; (**b**) trapezoidal; (**c**) trapezoidal cap; (**d**) stepped.

Finite-element simulations of cold extrusion were conducted for the four blank cross-sectional shapes shown in Figure 4. While the blank geometries differed, all other parameters in the finite-element models—including extrusion distance, extrusion speed, and friction coefficient were identical. All blanks were front orientation. The simulation steps required to achieve the 6.5 mm fin height on the copper substrate were selected for analysis. Figure 6 compares equivalent stresses across different shapes. Results indicate that among the four shapes, the stepped structure exhibits the lowest equivalent stress of 719 MPa. The equivalent stress values at five identical positions on the needle fins after forming were extracted for the four shapes, respectively. The results show that the stress values of the rectangular and trapezoidal cap-shaped fins are comparable, while the trapezoidal shape exhibits the highest stress value among its needle fins, peaking at 524 MPa. For the stepped shape, the maximum needle-fin stress reaches 500 MPa, with the remaining four measuring points all around 480 MPa. When the blank cross-section is trapezoidal or trapezoidal cap-shaped, the shape change in the transverse direction is continuous. During needle-fin forming, the transition zone also undergoes simultaneous deformation, i.e., in addition to material flowing from the top into the needle-fin holes, material from the transition zone also flows toward the needle-fin holes. Consequently, the final equivalent stress is relatively high. In contrast, the stepped shape employs localized geometric design. During forming, material from the upper part of the step primarily flows into the pinhole, and with relatively low constraints around the periphery, stress concentration is dispersed.

**Figure 7:**
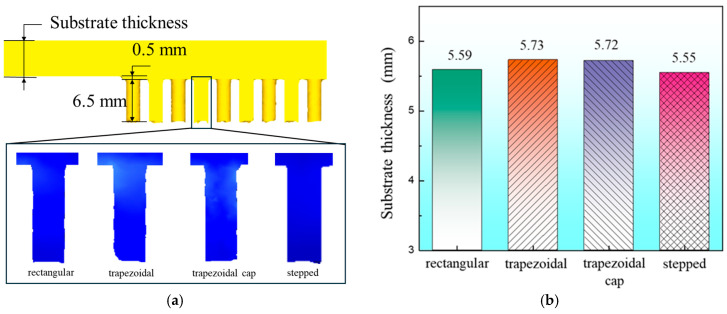
(**a**) Schematic diagram of substrate thickness measurement; (**b**) Substrate thickness after forming.

After cold extrusion forming of four blank cross-sectional shapes, the substrate thickness was measured when the needle-fin height reached 6.5 mm, as shown in Figure 7a. The substrate thickness results after forming with different shapes are shown in Figure 7b. The thickness of the rectangular copper substrate after forming is 5.59 mm. The thicknesses of the trapezoidal, trapezoidal cap, and stepped shapes are 5.73 mm, 5.72 mm, and 5.55 mm, respectively. The difference between the trapezoidal and trapezoidal cap thicknesses is 0.01 mm, which is negligible. Among the four shapes, the stepped blank exhibits the thinnest copper substrate base thickness after forming. Since the initial volumes are identical, the better the filling effect of the needle fins during forming, the thinner the base thickness will be. The same needle-fin formed from blanks with different cross-sectional shapes was selected to compare filling effects. As shown in Figure 7a, the needle-fin with a stepped cross-section exhibits the best filling effect, while those formed from trapezoidal and trapezoidal cap cross-sections display missing material defects around their peripheries. Therefore, the step-shaped blank achieves the best forming results.

**Figure 8:**
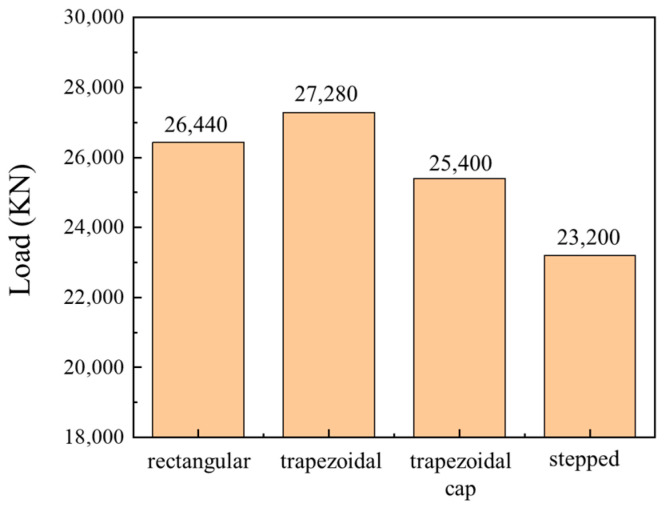
Forming loads for different-shaped blanks.

Reducing forming loads has always been a key objective in forming experiments. Figure 8 shows the forming load results for four blank shapes: the trapezoidal blank exhibited the highest load at 27,280 kN, the rectangular blank at 26,440 kN, the trapezoidal cap at 25,400 kN, and the stepped blank at the lowest load of 23,200 kN. Therefore, the stepped blank demonstrated the most favorable forming results.

The shape of the blank directly determines material flow behavior, stress distribution, and contact interface conditions. Rectangular blanks exhibit significantly greater initial contact area than needle-fin cavity surfaces, leading to pronounced localized stress concentration during the early extrusion stage. Material must overcome substantial flow resistance to fill the needle-fin cavities, increasing forming energy consumption. While trapezoidal blanks partially improve material flow direction through their inclined surface design, their contact area gradient remains steep. This creates shear stress peaks in the transition zone, hindering smooth material flow. Trapezoidal cap-shaped blanks further optimize top contact, but abrupt transitions persist between the bottom and needle fins, leading to uneven stress distribution and discontinuous material flow paths. In contrast, the stepped blank achieves a progressive distribution of contact area through its multi-level stepped structure. Hwang Y M et al. [26] found that when the extrusion conditions are (1) the geometric center of the fin part in coincidence with the center of the die and (2) a fully blocked die orifice at the base part, products with good forming results can be obtained. Compared to other blank shapes, the stepped blank minimizes the contact area with the die hole while satisfying both conditions. Kim K M et al. [27] observed that localized plastic strain in cold forging induces stress concentration, exacerbating work hardening, while uniform flow paths suppress excessive local deformation. In this study, the stepped design avoided shear stress peaks prone to occur in continuous geometric transition zones through discrete contact areas, thereby enhancing the uniformity of macroscopic plastic deformation. During the initial extrusion phase, material at the front of the steps fills the needle-fin cavity first and discontinuously, effectively reducing the local stress concentration factor. Simultaneously, its stepped profile minimizes radial shear and turbulence, enhancing material filling efficiency and forming stability. Therefore, under identical process conditions, the stepped blank achieves optimal filling results and the lowest required tonnage due to its more rational contact area distribution, lower stress concentration levels, and smoother material flow path, significantly reducing flow resistance and deformation work during extrusion.

### 3.2. Influence of Placement Methods on Forming Quality

**Figure 9:**
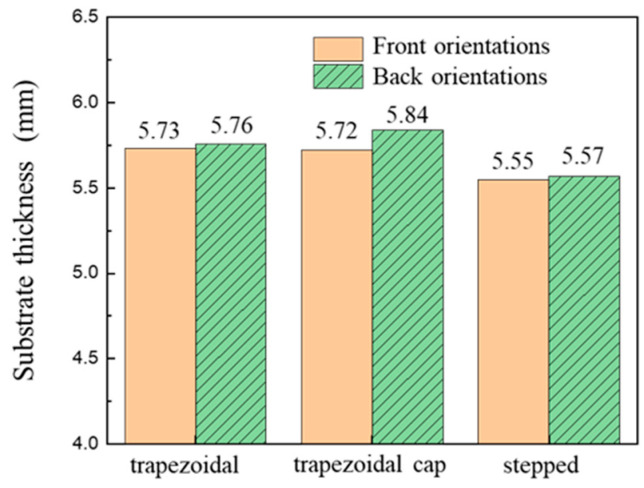
Forming substrate thicknesses for different-shaped and placement blanks.

Since there is no difference between the front and back placement methods for rectangular blanks, this shape is not studied in this section. After cold extrusion forming of three blank cross-sectional shapes in both front and back placement methods, the substrate thickness was measured when the needle-fin height reached 6.5 mm, as shown in Figure 9. In back orientations, the substrate thicknesses of the trapezoidal cap, stepped shape, and trapezoidal blank shapes were 5.76 mm, 5.84 mm, and 5.57 mm, respectively. All substrate thicknesses were greater than those in the front orientations, with the trapezoidal cap blank shape exhibiting the most significant increase at 0.12 mm. During the initial forming stage, the downward movement of the punch causes the material to undergo two deformations simultaneously: it extrudes the smaller-area end to form the base substrate while driving the material at the larger-area end to form the needle-fin structure. After the needle fins were fully formed, the punch continued forging until the upper plane was completely formed. Under these conditions, the filling effect of the needle fins was poorest. When the needle-fin height reached 6.5 mm, the substrate thickness was at its maximum.

**Figure 10:**
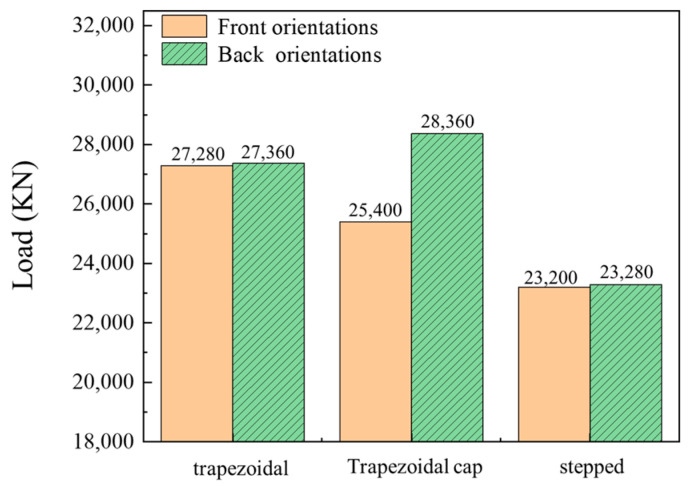
Forming loads for different-shaped and placement blanks.

Figure 10 compares forming loads for three shapes placed in both front and back orientations. Results indicate that forming loads with back orientations consistently exceed the loads with front orientations. However, the trapezoidal and stepped blank shape configurations show minimal load variation, whereas the trapezoidal cap blank shape exhibits a significant load difference of 2940 kN. In the front orientations, the smaller cross-sectional areas of all three blank shapes align with the needle-fin cavity die regions, enabling material flow dominated by low-resistance shear deformation. Conversely, the back orientations require filling a larger cavity with a smaller cross-section, necessitating higher loads to overcome interfacial constraints.

**Figure 11:**
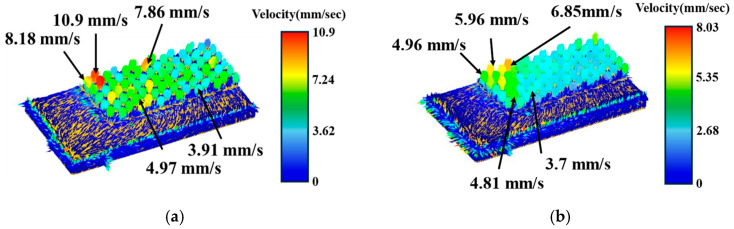
Material flow velocity of trapezoidal cap blank shape: (**a**) front placement; (**b**) back placement.

**Figure 12:**
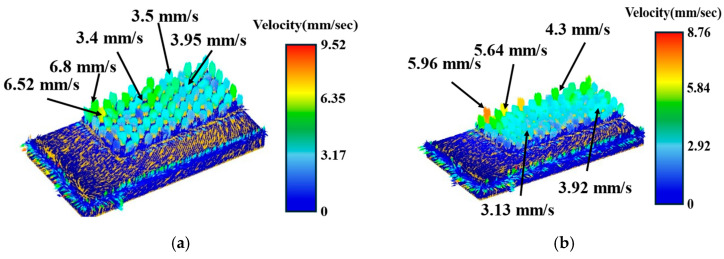
Material flow velocity of stepped blank shape: (**a**) front placement; (**b**) back placement.

Taking the trapezoidal cap and stepped blank shape as examples, the flow velocities under the front and back placement methods were compared. As shown in Figure 11 and Figure 12, the flow velocity contour plots reveal that the overall flow velocity was higher for both blank shape types when front orientations were compared to back orientations. For the trapezoidal cap-shaped blank, the peak flow velocity reached 10.9 mm/s in the front orientation, whereas it was only 8.03 mm/s in the back orientation. The step-shaped blank exhibited a peak flow velocity of 9.52 mm/s in the front orientation, which decreased to 8.76 mm/s in the back orientation. Simultaneously, the velocity distribution of the trapezoidal cap-shaped blank in the front orientation exhibits significant non-uniformity, with high-velocity zones (localized red areas) and low-velocity zones (blue areas) interspersed in the velocity contour map. In contrast, the velocity contour map of the step-shaped blank in the front orientation shows more consistent color distribution, combining high velocity levels with good uniformity. Tang D et al. [25] found that optimizing friction conditions during the extrusion process directly influences microstructural evolution and deformation uniformity. Volz S et al. [28] also observed that reducing the coefficient of friction in cold forging significantly decreases energy dissipation and improves macroscopic deformation uniformity. The step-shaped blank in the front orientation reduced friction resistance by decreasing the initial contact area, thereby promoting uniform material flow and further substantiating the positive impact of low friction on plastic flow stability.

All front orientation layouts demonstrated superior needle-column filling and lower forming loads, with the stepped blank shape exhibiting particularly outstanding results. Xue X et al. [24] found that in porous extrusion, the optimal matching of initial contact area is crucial for controlling material flow equilibrium, significantly reducing forming loads and enhancing filling uniformity. In this study, under front orientation, the cross-section of the blank directly corresponds to the needle-column forming zone. This provides superior initial cross-sectional matching for material supply, enabling more abundant and stable material flow momentum for the needle-column structure during extrusion. Consequently, resistance to material diversion and localized accumulation is reduced. The findings are consistent with the conclusions of Xue X et al. [24]. Meanwhile, the geometric characteristics of the stepped blank shape better align with the gradient demands of extrusion flow. Compared to the trapezoidal and trapezoidal cap blank shape, the stepped structure of their cross-sectional transitions further optimizes material flow paths and reduces velocity fluctuations. This simultaneously enhances filling efficiency, load characteristics, and flow velocity uniformity.

### 3.3. Final Blank Size Optimization

**Figure 13:**
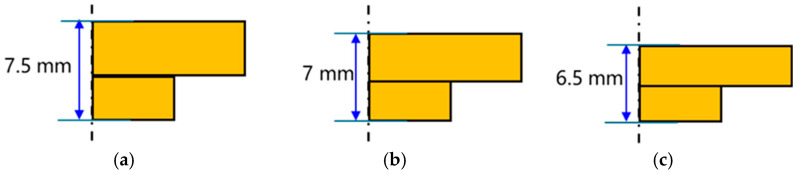
Stepped blank of varying thicknesses: (**a**) The thickness of the blank is 7.5 mm; (**b**) 7 mm; (**c**) 6.5 mm.

The results above indicate that the step-shaped blank with front orientation achieves the best forming results and the lowest forming load among the four blank types. To further reduce costs and improve material utilization, the dimensions of the step-shaped blank were optimized. The optimization approach involved reducing the overall blank thickness. Starting from the initial thickness shown in Figure 4d, the thickness decreased in increments of 0.5 mm. Other dimensions in the thickness direction are proportionally scaled, while the lateral dimensions and cross-sectional shape remain unchanged. Figure 13 illustrates the blank dimensions.

**Figure 14:**
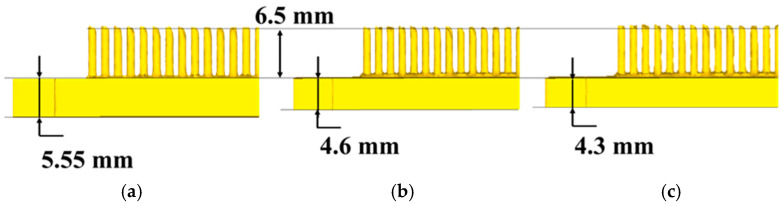
Finite element simulation results for extrusion forming of stepped profile blanks with varying thicknesses: (**a**) The thickness of the blank is 7.5 mm; (**b**) 7 mm; (**c**) 6.5 mm.

**Figure 15:**
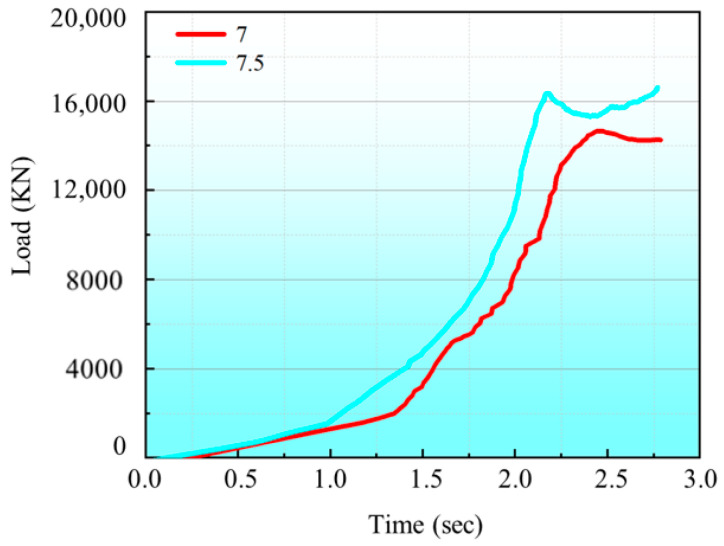
Forming load for different initial thicknesses.

Figure 14 shows the finite-element simulation results for cold extrusion forming of blank materials with different thickness step profiles. At an initial thickness of 6.5 mm, when the fin height reached the required 6.5 mm, the substrate thickness was 4.3 mm. This was less than the required 4.5 mm, thus failing to meet dimensional specifications. However, with an initial thickness of 7 mm, the substrate thickness of the formed part is 4.6 mm, with both base thickness and needle-fin height meeting product requirements. Moreover, material utilization is lower than when the initial thickness is 7.5 mm. Compared to the forming load at an initial thickness of 7.5 mm, as shown in Figure 15, the load decreased from 23,200 KN to 15,000 KN, representing a reduction of 35.3%. Therefore, the optimal shape and dimensions are those depicted in Figure 13b.

### 3.4. Experimental Validation

**Figure 16:**
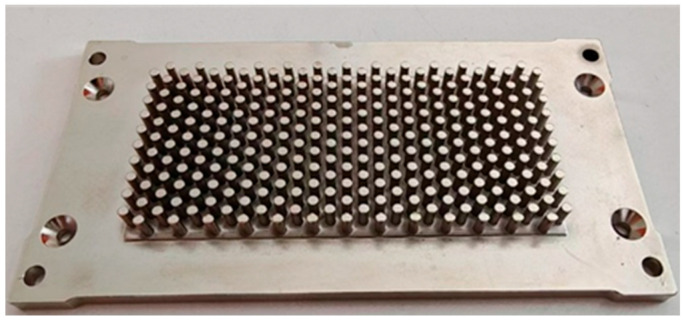
Finished part after cold forging and subsequent machining.

Based on the optimized results obtained from simulation, a 7 mm-thick stepped initial blank was selected for cold extrusion testing. Figure 16 shows the finished part after cold forging and subsequent machining. Measurements confirmed that all needle-fin heights reached 6.5 mm, with the copper substrate thickness at 4.5 mm, fully meeting specifications. The extrusion force required was 16,320 KN, showing an 8.8% deviation from the simulated results. This discrepancy was primarily due to the simulation’s overly idealized friction settings and its simplification of die and equipment deformation and fit, both of which result in higher actual forming loads. The feasibility of enhancing material utilization and reducing forming loads by altering the shape and dimensions of the blank has been demonstrated.

## 4. Conclusions

To enhance material utilization and reduce cold extrusion tonnage, this paper investigates the effects of different blank shapes, dimensions, and placement orientations on the final dimensions of cold-extruded copper pin-type heat sink substrate through finite-element simulation. The main conclusions are as follows:

(1) The blank shape has a significant impact on the cold extrusion forming effect of copper pin-type heat sink substrates. The stepped blank, with its multi-level structural design, achieves a progressive distribution of contact area, effectively dispersing stress concentration and reducing material flow resistance. Its forming quality and load characteristics are superior to those of rectangular, trapezoidal, and trapezoidal cap blanks.

(2) The blank placement method directly affects material flow and forming load. The front orientation of the three non-rectangular blanks is superior to the back orientation, enabling lower forming load, higher material flow velocity, and more uniform velocity distribution. Among them, the placement method of the trapezoidal cap blank has the most significant impact on the load.

(3) After dimension optimization, the 7 mm-thick stepped blank is determined as the optimal solution. Compared with the initial 7.5 mm-thick blank, the forming load is reduced by 35.3%, the material utilization rate is significantly improved, and the substrate thickness and pin-fin height of the formed part fully meet the design specifications.

(4) The finite-element simulation results are highly consistent with the experimental data, verifying the reliability of the simulation model and the engineering practicality of the optimized scheme, which provides a reference for the cold extrusion process optimization of similar heat sink substrates.

(5) The systematic design of four blank shapes with different structural characteristics and the proposal of the front/back placement orientation method for non-rectangular blanks, which reveals the intrinsic mechanism of blank structure on material flow, stress distribution, and forming load in the cold extrusion process of copper pin-type heat dissipation substrates. The optimized stepped blank cold extrusion process effectively solves the key engineering problems of low material utilization rate and high forming load in the traditional process of copper pin-type heat dissipation substrate forming. This study combines simulation optimization with actual production needs, and the obtained optimal scheme can be directly applied to the mass production of copper pin-type heat dissipation substrates, providing a practical and cost-effective technical path for the industrial production of such parts.

(6) This study has certain research limitations. In order to focus on the engineering application of the optimal scheme, only the stepped blank with the best forming performance was selected for cold extrusion physical experiment verification, and the complete experimental research on the forming process and forming parts performance of rectangular, trapezoidal, and trapezoidal cap blanks was not carried out. At the same time, there is a lack of comparative data on the microstructure, mechanical properties, and actual heat dissipation performance of formed parts from different shape blanks, which makes it impossible to carry out a more comprehensive and in-depth comparative analysis of the forming effect of various blanks. Future research will focus on the research limitations.

## Data Availability

The original contributions presented in this study are included in the article. Further inquiries can be directed to the corresponding authors.
